# Building a Brain Tumor Practice: Objective Analysis of Referral Patterns and Implications for the Growth of a Subspecialty Surgical Program

**DOI:** 10.7759/cureus.10416

**Published:** 2020-09-12

**Authors:** Daniel G Eichberg, Richard H Epstein, Franklin Dexter, Long Di, Jason D Vadhan, Evan Luther, Ricardo J Komotar

**Affiliations:** 1 Neurological Surgery, University of Miami Miller School of Medicine, Miami, USA; 2 Anesthesiology, University of Miami Miller School of Medicine, Miami, USA; 3 Anesthesiology, University of Iowa, Iowa City, USA; 4 Neurological Surgery, College of Osteopathic Medicine, Nova Southeastern University, Miami, USA

**Keywords:** brain neoplasms, neurosurgeons, neurosurgery, operating rooms, referral and consultation, appointments and schedules

## Abstract

Introduction

Growth of surgical caseload among specialties with a large contribution margin is an important financial objective for hospitals. In this study, we examined the diversity of referral patterns to a neurosurgeon over an eight-year interval and examined practice attributes related to surgical growth.

Methods

The electronic records of all patients undergoing an intracranial surgical procedure between August 2011 and August 2019 by an academic neurosurgeon were reviewed retrospectively. The Herfindahl-Hirschman index (HHI) was used to assess the distribution of referrals among community physicians who referred such patients; a value of HHI <0.15 indicates diversity. The yearly HHI trend was evaluated using meta-regression.

Results

The neurosurgeon’s brain surgery caseload progressively increased on an annual basis from 1.4 to 12.5 cases per week between 2012 and 2018. Among the 1540 cases referred by 1775 different physicians, 78% were from three counties in southeast Florida and 8.1% from two counties in southwest Florida. The HHI declined between 2013 and 2018 by 0.023 per year (0.0046 standard error [SE], p = 0.0073) with the estimated value 0.0063 (0.0014 SE) < 0.15 in 2018 (p < 0.0001). The findings indicate that the base of referring physicians was highly diverse and that growth in caseload was accompanied by significantly less concentration of referrals.

Conclusion

Surgical growth in the neurosurgeon’s practice resulted from a small number of referrals from many physicians, not from many referrals from a small number of physicians. Few physicians referred a sufficient number of patients to warrant attribution of the referral itself to personal knowledge of their patients' eventual outcomes. Rather, factors promoting timely access to patient care appear to have been the driving force for growth.

## Introduction

Overall growth in caseload at hospitals mostly occurs from many different surgeons doing a small number of cases each week [1] and is unrelated to the extent of diversification of physiologically complex surgical procedures [2,3]. For hospitals looking to increase their net income, growing surgical volume for service lines with a large contribution margin per hour of operating room (OR) time is a strategic objective [4,5]. There is substantial heterogeneity in relative hospital margin per OR hour (>20-fold) among surgical specialties [2], and among surgeons (100-fold) [6]. Approaches such as recruiting surgeons with national reputations and launching a marketing campaign to attract patients are sometimes followed. However, this tactic is constrained by the limited number of such individuals, high recruiting costs, considerable expenses to meet the physician's clinical and research requirements, and risk to the marketing plan from competing hospitals’ countermeasures. Thus, typically, in metropolitan areas with many patients, the key question for substantive practice growth by a relatively unknown surgeon is how to generate community referrals most efficiently.

In this study, we examined the referral patterns for a neurosurgeon (RJK) specializing in brain tumors who greatly expanded his practice over an 8-year interval. Because our main interest was growth in brain surgery, we focused on his new patients who underwent such procedures. Understanding the factors contributing to the successful expansion of his surgical practice may provide insight to other surgeons and hospital administrators who desire similar growth in surgical volume.

We assessed the diversity of the referring physicians for each year by calculating the Herfindahl-Hirschman index (HHI). The HHI is used to assess the diversity of species in ecology (where it is known as Simpsons index) [7], to assess market competition in business [8], and to evaluate growth in surgery [2,9,10]. An HHI between 0.15 and 0.25 represents a moderately concentrated market, and above 0.25, a highly concentrated market when the United States Department of Justice assesses potential anti-competitive effects of mergers [11]. Analogously, an HHI <0.15 among physicians referring patients to the neurosurgeon would represent a lack of concentration (i.e., diversity).

Our hypothesis was that the growth in the neurosurgeon’s surgical practice occurred from a few patients referred by each of many physicians, as opposed to many patients referred by a few physicians.

The implication of high diversity among referring physicians would be that focusing on a few individual physicians would not have been sufficient to achieve growth. Because high diversity implies a low volume of patients per referring physician, assessment of outcomes in their patients could not have been a driver of referrals. Rather, growth was based on accessibility to care.

## Materials and methods

The University of Miami Institutional Review Board approved this retrospective study (IRB #20160437) with a waiver of consent. The Strengthening the Reporting of Cohort Studies in Surgery guidelines (STROCSS 2019) were followed [12].

Data sources

We relied on an Excel worksheet (Microsoft, Redmond, WA) maintained by the neurosurgeon (RJK) for all his patients who underwent brain surgery between January 1, 2012, and August 1, 2019, at the University of Miami Hospital. The patient’s name, date of surgery, the procedure performed, referring physician, and referring physician’s postal code were reviewed. All patients had diagnostic imaging demonstrating an intracranial lesion before neurosurgical evaluation. The yearly number of new patients referred by each physician was tabulated. Surgical logs from the electronic health system (Epic Systems, Verona, WI) were examined to determine if a new patient underwent brain surgery within 365 days of the initial evaluation. Travel times were estimated using the Google Distance Matrix application programming interface (Google, Mountain View, CA) [13,14].

Attributes of the neurosurgeon’s practice

The various attributes the neurosurgeon implemented to encourage the growth of his surgical practice are described in Table 1.

**Table 1:** Attributes of the studied neurosurgeon’s practice related to surgical growth

Attribute of Neurosurgeon's Practice	Rationale
Frequent talks are given by the neurosurgeon at local medical society meetings and grand rounds at hospitals in the surrounding counties	Allows presentation of the scope of the neurosurgeon’s practice and his patients’ outcomes. Provides medical knowledge on the current treatment and management of patients with brain tumors.
Referring physicians are given information regarding the neurosurgeon's outcomes for the relevant brain tumor and are sent his relevant published papers.	Provides evidence of the neurosurgeon's ongoing scholarly activity and specific references may assist the referring physicians in subsequent discussions with their patients.
The neurosurgeon’s cell phone number is given to physicians, who were encouraged to call him if they desired assistance with a patient with an intracranial lesion.	Intracranial lesions represent urgent conditions that require immediate expert management. Direct contact facilitates the rapid evaluation of these patients and the transmittal of diagnostic imaging.
All new patients must have diagnostic imaging showing an intracranial lesion before scheduling.	Workup is initiated by the referring physician, and thus patients without an intracranial lesion are screened out ahead of time.
Patient contact information is obtained by the neurosurgeon and forwarded to his outpatient scheduler. The scheduler then contacted the patient and booked the clinic appointment.	University hospital scheduling phone lines can be frustrating to patients due to delays in answering, waiting for callbacks, and undesired constraints imposed related to the presence of “open” appointment slots.
All new patients are offered an outpatient appointment within 48 hours.	Patients are seen even on days when the neurosurgeon has scheduled operating room time (3-4 days a week). Patients are seen in between cases or at the end of the day and in fewer than 48 hours if clinically indicated. If the patient prefers a longer interval to the appointment, that is accommodated.
Personal follow-up is made by the neurosurgeon with the referring physician.	The referring physician is up to date on their patient’s medical treatment and able to provide better continuity of care
Patients are sent back to their referring physician for subsequent care.	Ensures continuity of care and continued collaboration in the future.

Statistics

The HHI was calculated as the sum of the squares of the proportions of cases from each referring physician. The inverse of the HHI is the effective number of items measured (e.g., species, businesses, physicians) [9,15]. As a simple example, consider four physicians referring 4, 3, 2, and 1 cases, N=10 cases; HHI = (0.42 + 0.32 + 0.22 + 0.12) = 0.31. The effective number of referring physicians = 3.23 (1/0.31). 

The binomial lower 95% confidence intervals for proportions of cases were calculated using the method of Clopper-Pearson. The fraction of new patients who had surgery within 365 days of their initial evaluation was calculated by batching among 4-week periods and reported as the mean (95% confidence interval [CI]). The standard errors of the HHI and the inverse of the HHI were estimated asymptomatically for each year with full data [9]; all years had at least 67 cases referred by physicians. The trend in the HHI was assessed by testing the slope of the regression line, using the corresponding t-distribution, with the STATA function meta regress (STATA, College Station, TX). Values for the HHI and the effective number of referring physicians are presented as the mean (standard error). As this was a descriptive study, and all patients were included, no a priori power analysis was performed.

## Results

**Figure 1:**
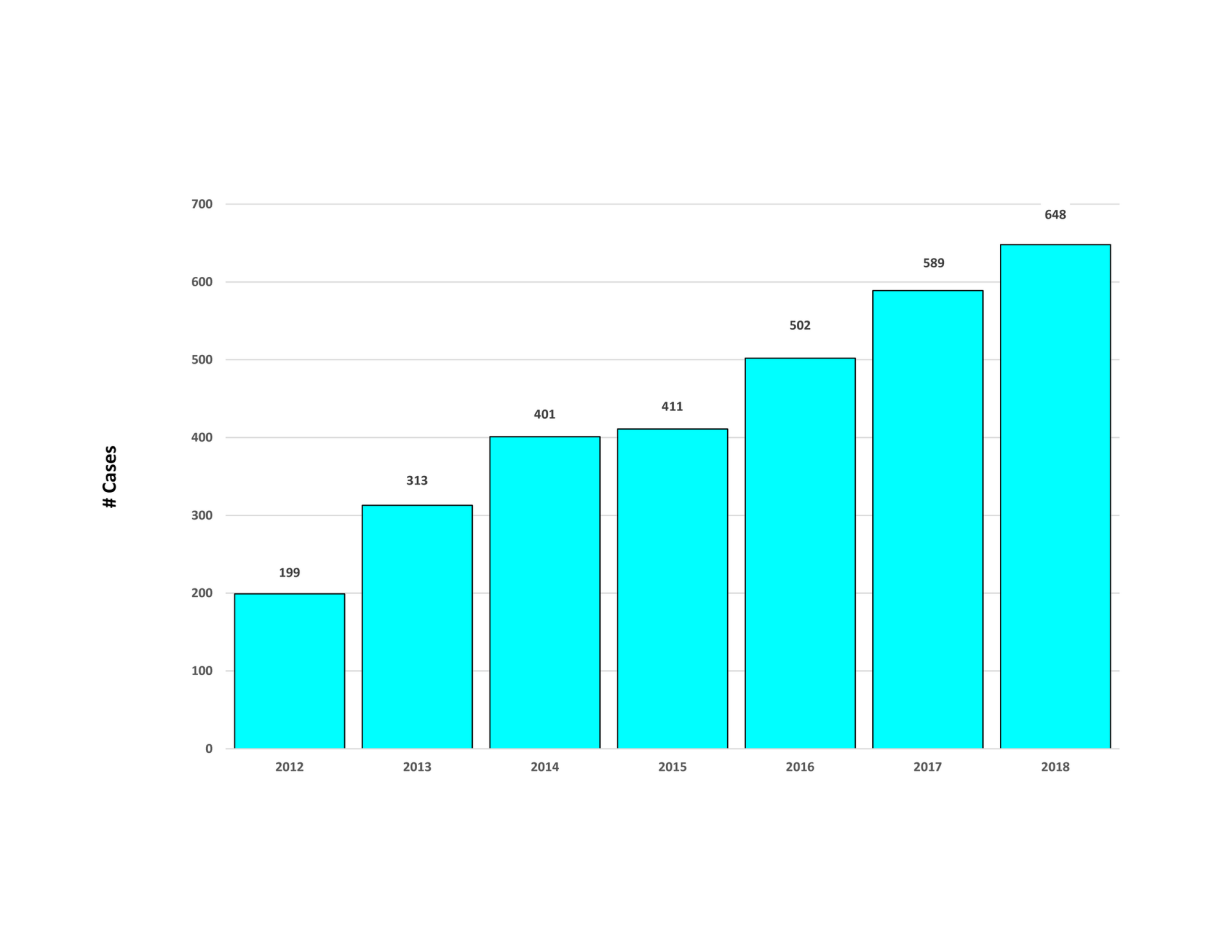
Annual caseload of brain tumor surgery by the studied neurosurgeon between January 1, 2012, and December 31, 2018. Cases from 2011 are not shown because the neurosurgeon started operating in August 2011. Cases from 2019 are not shown because the data analyzed only included cases through July 2019.

Between August 1, 2011, when the neurosurgeon began operating at the hospital, and December 31, 2018, the total number of his brain surgery cases increased from 1.4 to 12.5 per week (Figure 1). The cases included both intracranial procedures and radiosurgery. Among patients referred to the neurosurgeon between January 1, 2018, and December 31, 2018, he performed an operative procedure in 54.1% (95% CI 50.3% to 57.9%) within the next 365 days (median 6 days, interquartile range 3 to 12 days).

**Figure 2:**
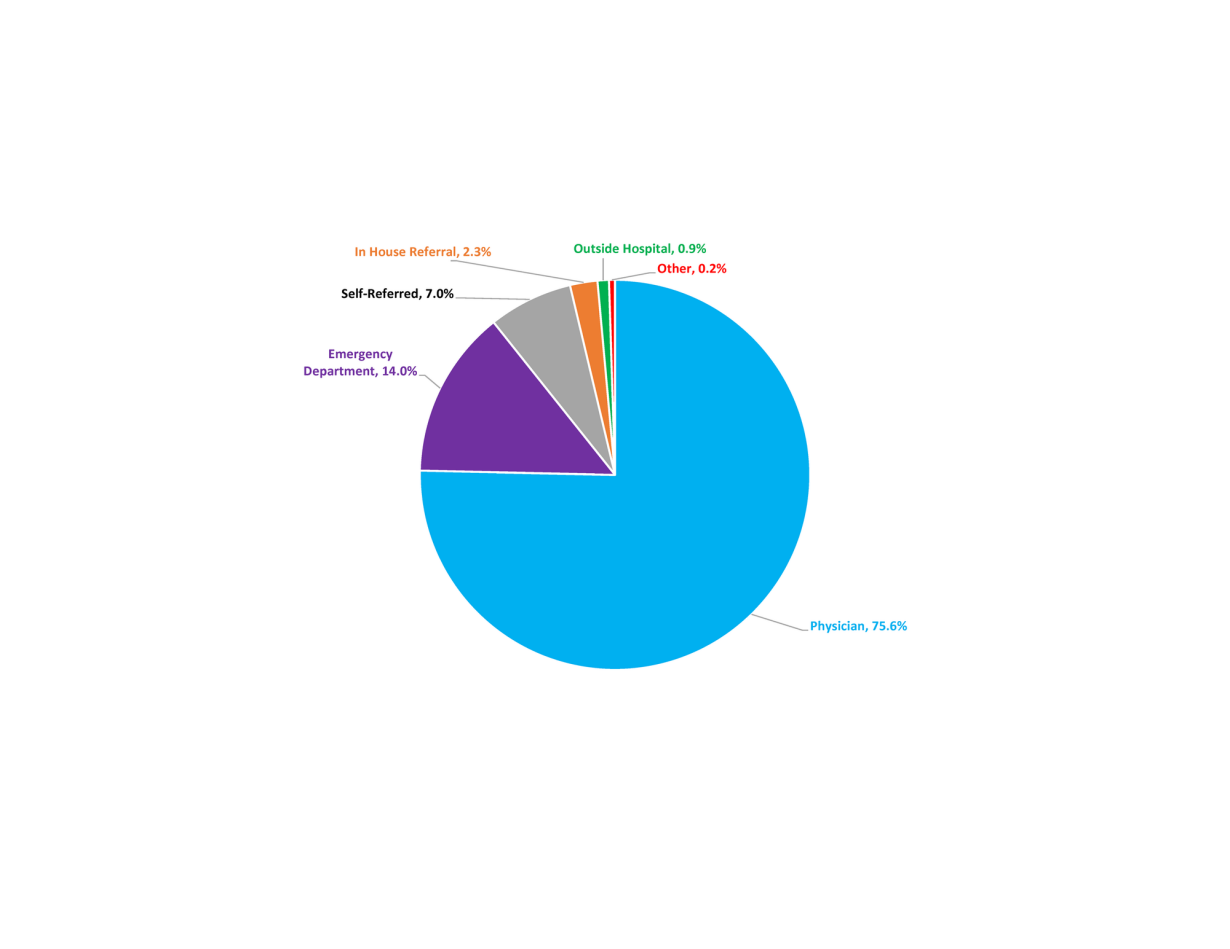
Sources of referrals to the neurosurgeon’s practice for patients who underwent a surgical procedure, January 1, 2012, to August 1, 2019.

Approximately 75% of the neurosurgeon’s operative practice was from outside referrals, highlighting this source’s importance (Figure 2).

**Figure 3:**
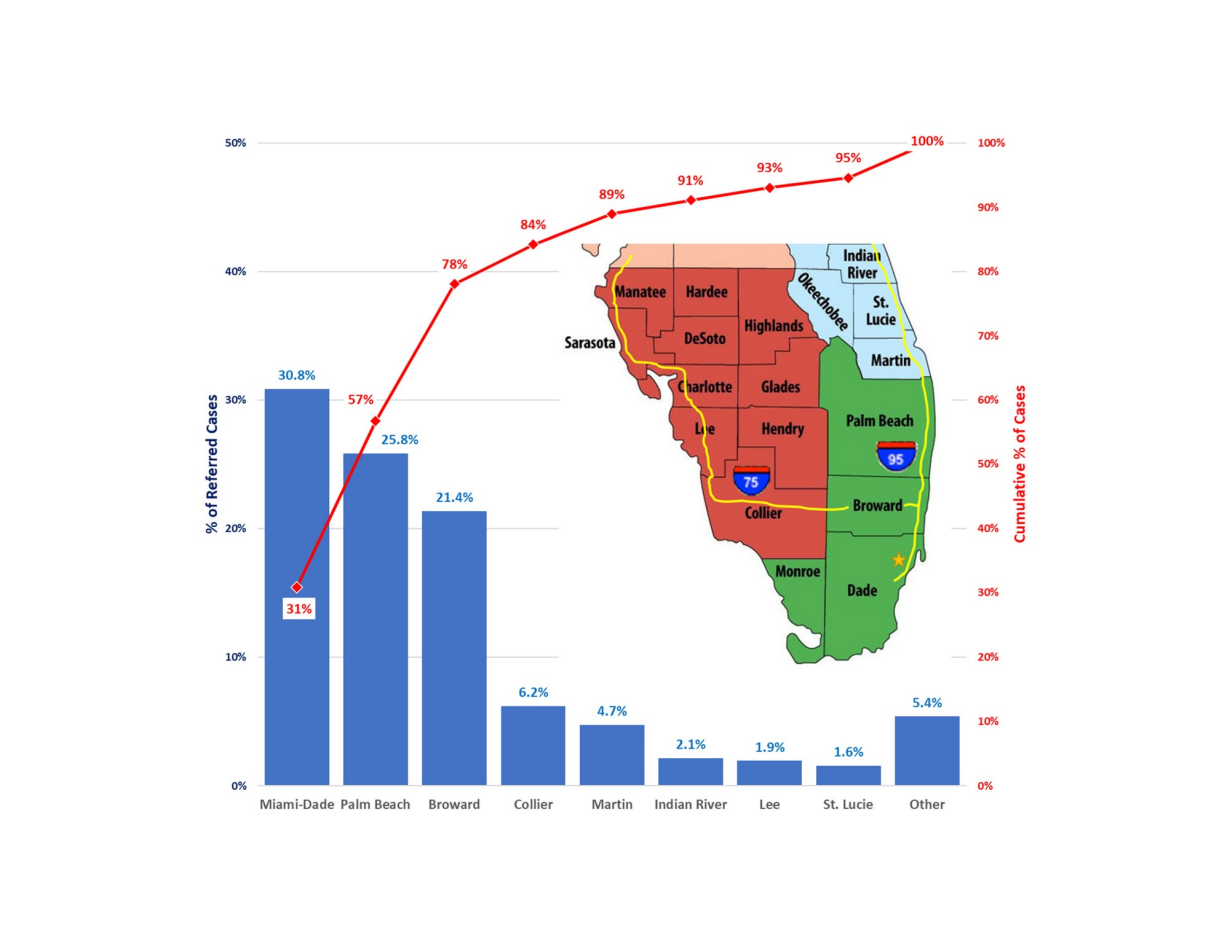
Geographic location, by county, of community physicians referring surgical patients to the studied neurosurgeon, and the approximate position of the relevant interstate highways. Referrals from inside the hospital where the neurosurgeon practiced were excluded. The map of southern Florida is overlaid on the graph, with the gold star indicating the approximate position of the University of Miami Hospital, the practice location of the studied neurosurgeon. Interstate-95 passes within 1 mile of the hospital and follows the eastern coast of Florida north through Broward, Palm Beach, Martin, St. Lucie, and Indian River Counties. Interstate-75 runs north to south through Lee and Collier counties, then east through the middle of Broward County, where it connects with Interstate-95.

Of cases referred by an outside physician, 78% were from three counties in southeast Florida (Miami-Dade, Broward, and Palm Beach), and 8.1% from two nearby counties in southwest Florida (Collier and Lee) (Figure 3). There is high-speed interstate highway access from these counties, located within a 2.25 hour driving time from the hospital (Figure 3).

**Figure 4:**
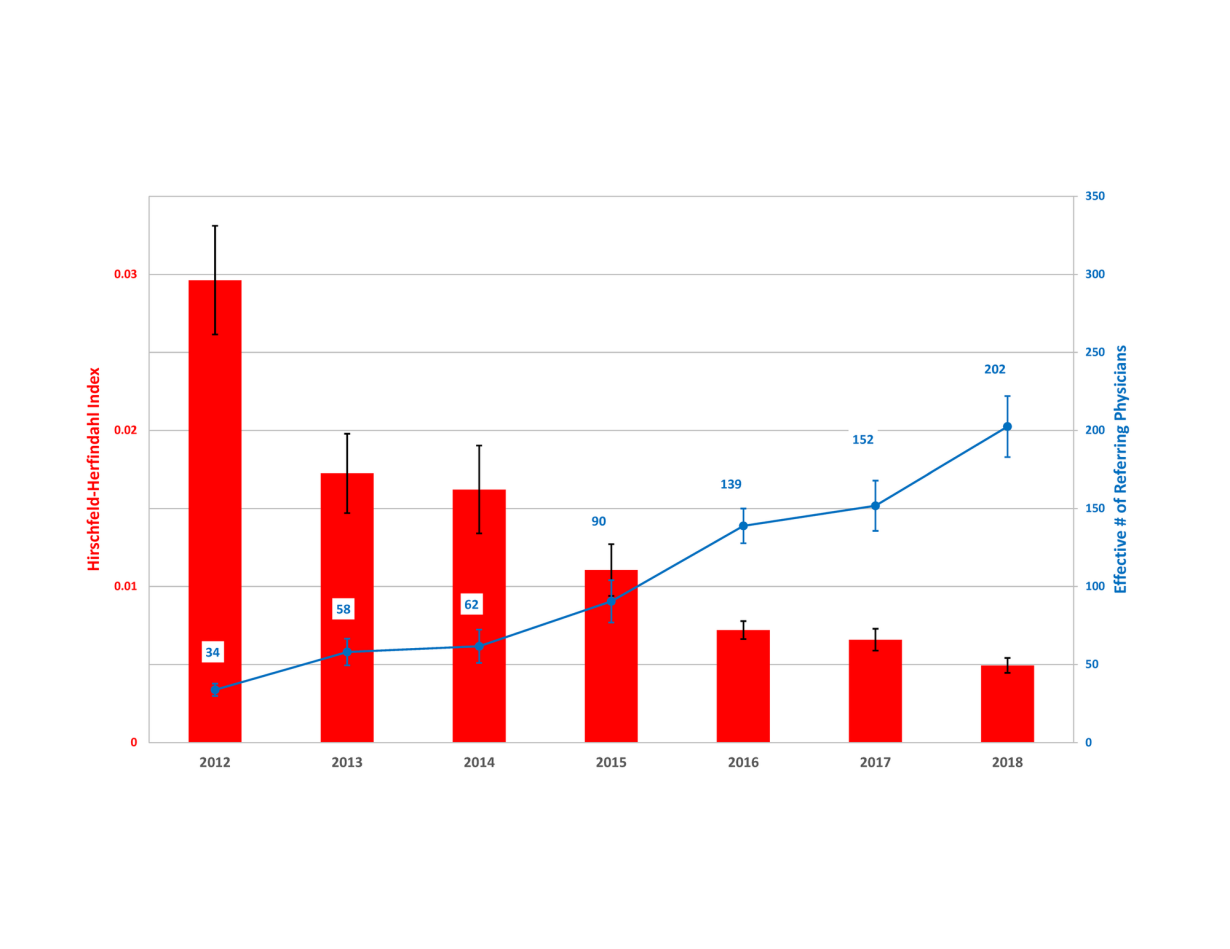
Market concentration of physicians referring patients requiring surgery to the neurosurgeon. The Herfindahl-Hirschman index (HHI) is a measure of the diversity of the referring base of physicians (see Methods), and the effective number of referring physicians is calculated as 1/HHI. The lower the HHI, the greater the diversity in the number of referring physicians. Markets are considered to be diverse if the HHI is less than 0.15, 30-times larger than the neurosurgeon’s referral base of physicians in 2018 (0.0049 ± 0.00048 SE).

For each year with 12-months data (2012-2018), the HHI for referring physicians was < 0.03 and declined each year (Figure 4). These findings indicate a highly diverse base and no referring physician responsible for a substantive proportion of referrals. In 2018, the effective number of referring physicians was 202.4 (19.6), also showing the absence of market concentration. Each year was associated with a decline in the HHI by 0.0033 (0.00055) units (p = .0019). Treating the year 2018 as the intercept, the estimate of the HHI was 0.0056 (0.0019), significantly less than 0.15 (p < 0.00001). The decreasing value of the HHI over time shows that the growth in caseload was accompanied progressively by significantly less concentration of referrals among physicians.

**Figure 5:**
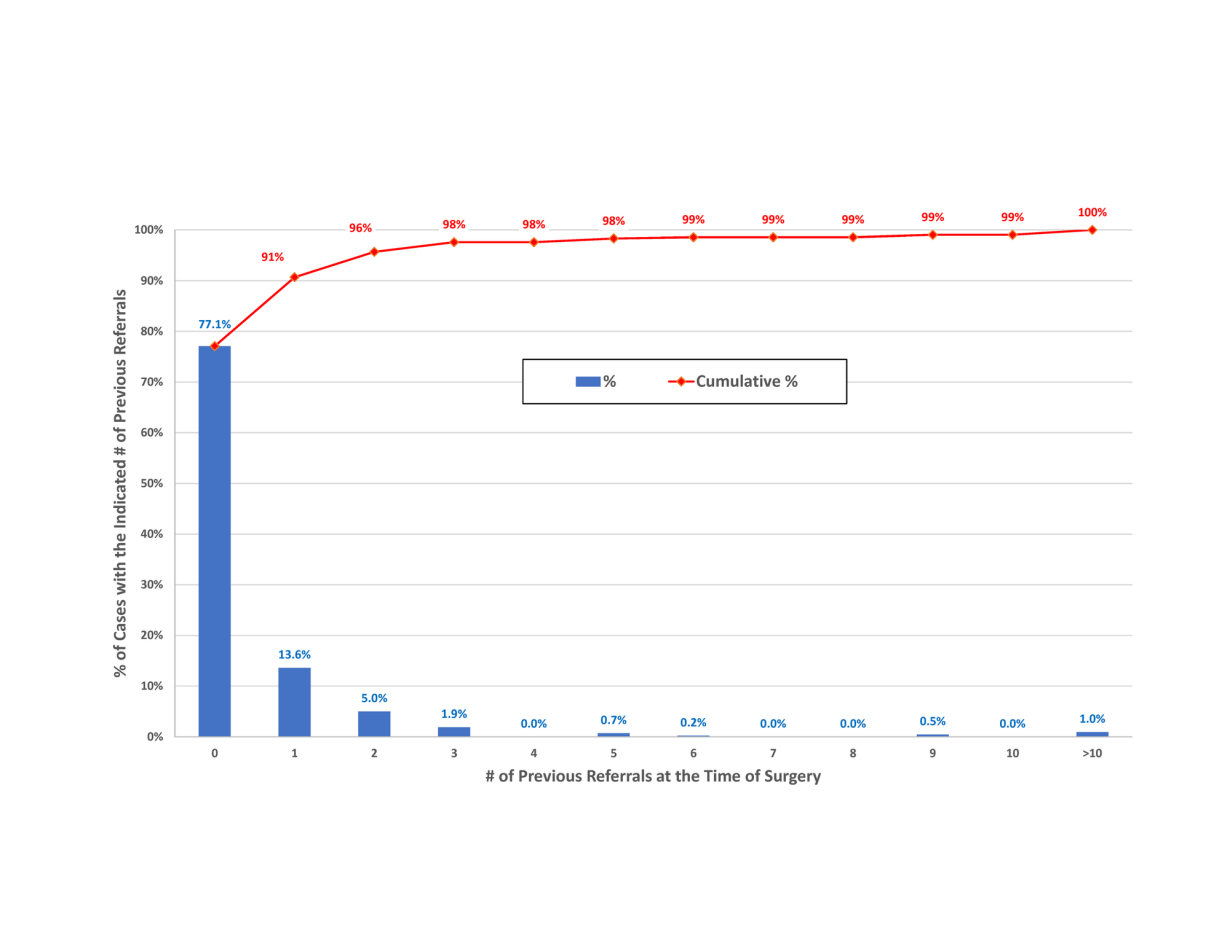
Referrals to the neurosurgeon within the previous year for patients undergoing surgery. For each case, the number of patients previously referred within 365 days by the referring physician for the current case was determined. For 71.1% of cases, the referring physician had not referred any previous patients, and for 13.6%, only one case had been referred. The 95% lower confidence limit for the percentage with zero or one referral within the previous 365 days was 87.5%.

Among the neurosurgeon’s cases between January 1, 2018, and August 1, 2019, 91% (87.5% lower confidence limit) of the cases were referred by a physician who had only referred 0 or 1 patients in the preceding 365 days (Figure 5).

## Discussion

Our results show that the neurosurgeon’s increase in surgical caseload was not a function of a small number of physicians referring many patients but rather from many physicians each referring a few patients. The vast majority of physicians only referred zero or one patient who had had surgery within the previous 365 days. Our finding implies that few physicians could have had a sufficient sample to use their own patients’ surgical outcomes to influence their decision to refer patients to the neurosurgeon.

There are substantive OR management issues related to providing rapid patient access to neurosurgical evaluation. First, committing to seeing patients within 48 hours means that time must be carved out during surgical days. A small delay in starting the next case because the surgeon is evaluating a new patient with a brain mass should be balanced by the high percentage who will need surgery. The variable cost of several additional minutes of turnover-time [16] is small compared to the hospital’s contribution margin from a brain surgery case. Anesthesiologists and OR managers should accommodate the surgeon to facilitate expedient patient evaluations. Second, seeing new patients within 48 hours did not increase the number of add-on cases. Rather, three-quarters of cases were scheduled over the ensuing 3 to 12 days.

Our finding of infrequent referral of patients by individual physicians is consistent with the study of Mandl et al., who showed that collaboration of patient care among all types of providers for outpatient clinic appointments was uncommon [17]. Our focus on the referring physician in the context of the treatment of a brain tumor seems appropriate based on the study of Charlton et al., who demonstrated that most rectal cancer patients relied on the advice of their own physician, and few attempted to assess either surgeon volume or experience in deciding where to seek definitive treatment [18]. Our finding that a substantive number of patients (e.g., from Miami-Dade, Palm Beach, and Broward counties) drove past many other hospitals offering brain surgery or traveled to Miami (e.g., from Collier and Lee counties) rather than to closer hospitals in the opposite direction is consistent with the study of Dexter et al., who showed that absolute distance rather than relative distance was the important travel consideration [19]. Based on previous survey studies indicating that patients focus more on the reputation of the surgeon than on that of the hospital [20-22], it is likely that the neurosurgeon’s practice growth was more related to his personal marketing efforts (e.g., hospital and medical society speaking engagements, dissemination of his personal cell-phone number) rather than the hospital’s general marketing campaigns. The extremely short waiting period (within 48 hours) for evaluation by the neurosurgeon (Table 1) likely played a major role in the expansion of his practice [23]. We do not have data related to the influence on referrals from the neurosurgeon sending his relevant outcomes publications [24-29] to referring physicians (Table 1), but we suspect that this also had a positive impact. In addition, the neurosurgeon is a deputy editor for the Cureus academic channel of the Department of Neurological Surgery at the University of Miami (https://www.cureus.com/channels/umiamineurosurg).

Our study has several implications for hospitals looking to increase surgical service line volume from neurosurgery or other subspecialty practices. First, adequate OR and clinic time must be allocated, with resources provided for efficient care. If the surgeon needs to evaluate a new patient between cases, the OR manager should focus on increasing the surgeon’s overall productivity rather than fixating on daily operational objectives. Such a perspective will also increase the overall productivity of the OR. Second, there needs to be flexibility in new patient appointment scheduling. Rigid systems requiring that patients go through central scheduling are not conducive to expeditious access, especially when all immediate appointments are booked or the surgeon is not scheduled for the clinic on a particular day but nonetheless is available to see new patients [30]. Marketing efforts to promote the surgeon’s practice largely should be focused within the distance that patients are willing to travel for care and should concentrate primarily on the expertise and outcomes of the surgeon, not on general attributes of the hospital.

Strengths and limitations

First, this study is from a single neurosurgeon’s practice involving a pathologic condition (i.e., brain tumor) that requires evaluation within a short time frame due to the risk of clinical deterioration. Thus, the window to see new patients probably can be extended for less urgent conditions. Nevertheless, patients do not want to wait very long for surgical evaluation, especially when dealing with possible or known malignancy. Second, all new patients seen had a brain mass on imaging before being scheduled, increasing the likelihood of surgical treatment (at least 50.3% in the studied neurosurgeon’s practice). Thus, making time to see patients even during busy days in the OR had a high yield. This strategy may differ for other subspecialties where surgery results less frequently following consultation. Third, referral records were not available for patients in whom a surgical intervention did not occur. However, we were primarily interested in growth in the neurosurgeon’s surgical practice.

## Conclusions

Expansion in the surgical practice of a neurosurgeon over an eight-year period was the result primarily of a small number of referrals from many physicians rather than many referrals from a small number of physicians. Physicians referring patients who required surgical intervention seldom had referred another such patient within the previous year. Timely access to care (seeing new patients within 48 hours), direct communication with referring physicians, and the ability to see new patients on any day, even when operating, appeared to be key factors associated with growth.
